# Beliefs about others’ intentions determine whether cooperation is the faster choice

**DOI:** 10.1038/s41598-018-25926-3

**Published:** 2018-05-14

**Authors:** Juana Castro Santa, Filippos Exadaktylos, Salvador Soto-Faraco

**Affiliations:** 1 Center for Brain and Cognition, Universitat Pompeu Fabra, Barcelona, 08002 Spain; 2 Department of Economics, Middlesex University Business School, London, NW4 4BT UK; 3 School of Agriculture Policy and Development, University of Reading, Reading, RG6 6AH UK; 4 Laboratory for Comparative Social Research, National Research University Higher School of Economics, Moscow, Russia; 5 Institut de Ciencia i Tecnologia Ambientals (ICTA), Universitat Autonoma de Barcelona, Barcelona, 08193 Spain; 6 Institució Catalana de Recerca i Estudis Avançats, Barcelona, 08010 Spain

## Abstract

Is collaboration the fast choice for humans? Past studies proposed that cooperation is a behavioural default, based on Response Times (RT) findings. Here we contend that the individual’s reckoning of the immediate social environment shapes her predisposition to cooperate and, hence, response latencies. In a social dilemma game, we manipulate the beliefs about the partner’s intentions to cooperate and show that they act as a switch that determines cooperation and defection RTs; when the partner’s intention to cooperate is perceived as high, cooperation choices are speeded up, while defection is slowed down. Importantly, this social context effect holds across varying expected payoffs, indicating that it modulates behaviour regardless of choices’ similarity in monetary terms. Moreover, this pattern is moderated by individual variability in social preferences: Among conditional cooperators, high cooperation beliefs speed up cooperation responses and slow down defection. Among free-riders, defection is always faster and more likely than cooperation, while high cooperation beliefs slow down all decisions. These results shed new light on the conflict of choices account of response latencies, as well as on the intuitive cooperation hypothesis, and can help to correctly interpret and reconcile previous, apparently contradictory results, by considering the role of context in social dilemmas.

## Introduction

Human cooperation –sacrificing individual resources to achieve higher collective welfare– poses an evolutionary puzzle in both social and natural sciences: even if selfishness leads to higher evolutionary fitness, cooperation is nevertheless widespread among humans^1–3^. Understanding the cognitive processes underlying human cooperation is essential for solving this puzzle. Researchers have therefore studied the Response Times (RT) of choices in social dilemma decisions (i.e. situations where private and social interests are in conflict) to address whether cooperation is the fast, unconflicted choice among humans (please note that quick choice speed has been taken to indicate the default or intuitive behaviour^4–7^, although this interpretation is not always conclusive). However, this method has produced mixed results; while several studies indeed found that individuals make cooperation choices significantly faster than selfish choices^4–8^, others reported the opposite pattern^9–11^. Similar findings were found employing different methodologies such as time pressure^7,12–14^, ego depletion methods^15–18^ as well as structural and functional neural correlates^19–22^; while most of the studies find that cooperation is the default choice among humans, other studies either fail to replicate or even find results in the opposite direction (for neuroeconomics see^23,24^; for time pressure^25–27^; for ego depletion^28^).

Taken together, the literature leaves little room for doubt that, at least in some occasions, cooperation is the choice favoured by individuals, or at least the one they take faster. The key question is then: Under which circumstances cooperation emerges as the unconflicted, fast choice? It has been suggested that RTs are driven by the overall attractiveness of the available choices: the more similar the expected payoffs of the available options are, the higher the conflict of the choice and ultimately the slower the RTs, since the choice is harder to make^29–32^. However, attractiveness is measured in units of utility, and therefore it may arise from a variety of factors including stimulus related factors (i.e. monetary returns of choices)^29^, individuals’ social preferences^21,29^, or evaluation of partners’ past behaviour^33,34^. As such, the conflict of choices account can explain some fast behaviours without the need to resort to intuition, but provides only limited information regarding the ultimate causes of cooperation speed. Here, we contend that within the conflict of choices paradigm, the social environment where the interaction takes place is most crucial in determining when cooperation will be the fast choice.

In particular, the present study focuses on the beliefs that decision makers assign to the intentions of other agents in a social situation. Judging the intentions and mental states of other individuals constitutes an integral part of everyday life human decision-making process, whereby the decision-maker evaluates reality before making a choice^35,36^. This is one of the most basic social inferences people make, and they can occur in a split-second^37^. In that way, beliefs about others’ intentions shape the social environment within which interactions take place and, consequently, they can moderate the behavioural choices^38–43^. For example, a person will often pass on offering help when seeing someone let an object fall on purpose, instead of accidently dropping it; the beliefs about the perceived intent in the first case suggests an environment where help is not necessary^44^.

In the case of social dilemmas, the beliefs about other’s intentions correspond to the perceptions of an individual regarding another individual’s cooperativeness, i.e. her intention to cooperate. Attributing to the other party a high chance of being cooperative might create an immediate social context where cooperation is the unconflicted, fast (perhaps default) choice. On the contrary, everything else equal, believing that the other party is unlikely to cooperate might give rise to defection being the choice more at hand at that occasion. Under such a framework, we hypothesize that whether cooperation will be the unconflicted, fast choice in a social dilemma situation will depend on the social environment which is set by individual’s cooperation beliefs about other agents.

The importance of the social environment emerging from cooperation beliefs in determining the speed of cooperation has been documented before. In^33^ and^34^ researchers re-analysed data from previously published studies of repeated Public Good and Prisoners Dilemma games and found that cooperation (defection) decisions were faster whenever the interacting partners had cooperated (defected) in the previous round. Similarly, in^45^ the authors reported that reciprocal choices of trustees in a strategy-method Trust game were faster. These results show clearly that cooperation beliefs affect when cooperative choices are unconflicted and fast.

However, it is not entirely clear whether beliefs in the above-mentioned studies affected the RTs by shaping the general social environment (defining it as cooperative or not) or by determining the payoff maximizing action of a particular round following a tit-for-tat strategy whereby cooperation (defection) is the best response after the other party chose to cooperate (defect) in the previous round. In particular, the use of fixed payoff-structure in the above designs may have affected the relative RTs of cooperation and defection decisions. For example, cooperation decisions were faster than defection ones in the original study of^7^, but the pattern reversed when the expected payoffs were changed so that the defection choices had even higher monetary returns in^29^. Therefore, to ensure that the social environment created by beliefs regarding intentionality is important for determining RTs, its effect should be observed across various levels of relative attractiveness of cooperation vs. defection choices in terms of expected monetary payoffs.

Additionally, individuals’ social preferences might also affect cooperation vs. defection RTs in a social dilemma situation. In^8^ and^46^ for example, it is shown that cooperation is the fast choice for pro-social but not for selfish (pro-self) individuals. Further, in^21^ the authors showed that whenever pro-social individuals take longer to decide they also tend to move toward more selfish choices, suggesting that these changes are due to a fear of being exploited; In contrast, selfish individuals move toward more cooperative choices. Importantly, these individual differences point to a potential interaction between one’s beliefs regarding other’s intentions and one’s social preferences: a belief that the partner will cooperate might create a cooperative social environment where cooperation is the preferred and fast option for pro-social individuals. In contrast, for selfish individuals, the very same (high cooperation) belief might instead be seen as an opportunity to take advantage of and free ride, making therefore defection the fast option. Indeed, in^33^ the authors report that the effect of cooperative (non-cooperative) beliefs was pronounced among individuals who chose to cooperate (defect) themselves in the previous round.

To address the framework laid out above we designed an experiment where we measured the *net* effect of beliefs regarding others’ cooperation intentions on choice RTs. In a social dilemma game, we experimentally manipulated the information the participants received regarding the cooperation behaviour of their current co-player, while simultaneously varying the expected payoffs of cooperation and defection choices. Participants made a series of 100 binary choices (following 5 training rounds) between a cooperative and a selfish option, with the latter always corresponding to higher expected payoffs. Crucially, the experimental environment was designed to minimize possible strategic considerations arising from the repeated game iterations: participants were told that their partner was different and randomly chosen in each round. In reality, there were no real co-players and participants played against predefined strategies with no feedback given between rounds.

**Figure 1:**
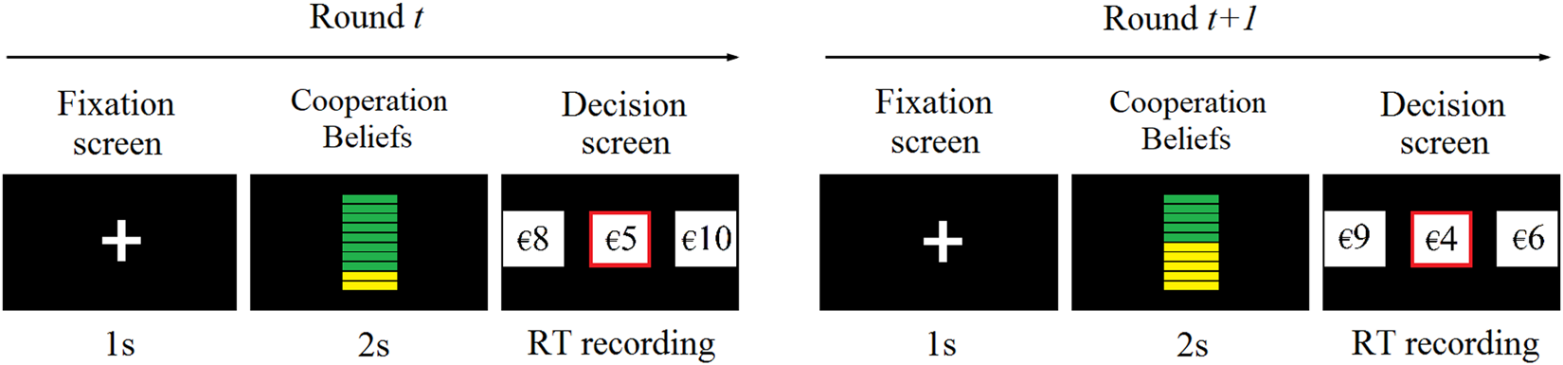
Schematic trial sequence. The figure shows two consecutive trials. Participants were presented with 100 such trials (in addition to 5 training rounds). After a fixation screen, which warned of the impending round and made sure participant’s gaze direction was consistent across the different trials, they were presented with a representation of the overall partner’s game strategy for two seconds. Subsequently, participants were presented with the decision screen which comprised the two choices (a low and a high monetary value, presented on the left and right), plus a penalty/reward value (in the centre). Choosing the low value always led to higher expected monetary returns and corresponded to defection strategy. Choosing the high value was the cooperative strategy. RTs were recorded from the presentation of the decision screen until the moment the participants pressed the key denoting their decision.

At the beginning of each round, participants were presented with the cooperation likelihoods of that rounds’ partner, in order to induce the corresponding belief in the participant, followed by the expected returns from cooperation/defection in that trial (Fig. 1). Information about the co-player’s cooperativeness was presented at the beginning of each round before presenting the payoffs, thus setting the social environment (i.e., beliefs) right from the beginning of each round. Cooperation beliefs were operationalized as the fraction of cooperative choices that the round’s co-player had predetermined to play for a total of 20 rounds (average probability of cooperation over 20 rounds, 0%, 20%, 50%, 80% and 100%). That is, beliefs could not be based on the previous rounds experience. Additionally, having five levels of beliefs about partner’s cooperativeness allowed more accurate estimation of parameters in the econometric analysis.

We analysed a total of 4600 decisions (46 participants × 100 decisions) and employed a random-effects model to estimate response latency (log(RT)) as a function of cooperation beliefs and expected payoffs, while controlling for other potential sources of variability (see Methods). This allowed us to estimate the net effects of beliefs on the RTs (Model 1, see Methods). Additionally, we also studied the effect of individuals’ social preferences by measuring participants’ cooperative behaviour based on their decisions across all rounds. We estimated the effect of social preferences on RTs (Model 2) and importantly, social preferences’ moderating effect (Model 3) by adding the interaction between social preferences and beliefs regarding the co-player’s cooperation intentions in the model (social preferences x cooperation beliefs).

## Results

**Figure 2:**
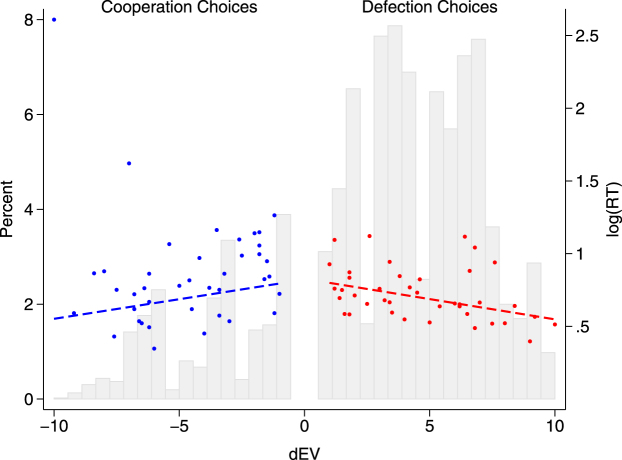
The effect of similarity in expected monetary payoffs on RTs of cooperation and defection choices. The dots represent the observed individuals’ mean log(RT) (right y-axis) as a function of dEV (i.e. expected value of the chosen option minus the expected value of the unchosen option), separately for cooperation and defection decisions (blue, red respectively). Dotted lines represent the prediction from the regression Model 1 (see text). The background light gray bars are the histogram of dEV, expressed as a percentage (left y-axis). Note that dEV takes negative values for cooperation decision and positive values for defection decisions. Therefore, for both cooperation and defection choices, the more similar the available option, the higher the RTs.

Overall, participants chose to cooperate 21.2% of the time, and the latency of cooperation choices (mean = 2.97 sec, sd = 3.32) was longer on average than that of selfish choices (mean = 2.65 sec, sd = 2.56; *z* = 4.69, *p* < 0.001). To study the role of similarity of choice in monetary payoffs, we used the difference in expected value (return) between the chosen and the unchosen option, which we denote as dEV (for a similar methodology see^29^). Our data confirms previous results: similarity of options in monetary terms drives RTs up. As seen from Fig. 2, mean observed response times are on average higher, the closer dEV is to zero, a point indicating equal payoffs between cooperation and defection options in expected return. The regression analysis confirms the result (see Model 1 in the Methods section and Table S1 in the Supplementary Information): everything else equal, the effect of dEV on logRT is positive for cooperation choices (*b* = 0.036, *p* < 0.001) and negative for defection ones (*b* = −0.018, *p* < 0.001). The different signs of the coefficients mirror actually the same effect: the more similar the options were, the higher the RTs. The linear prediction of the model controlling for cooperation beliefs and other confounding factors is also depicted in Fig. 2 (dashed lines).

**Figure 3:**
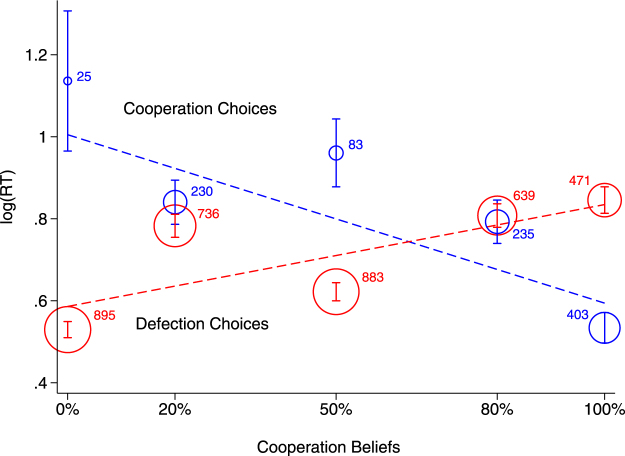
The effect of cooperation beliefs on RTs for cooperation and defection choices. The position of circles represents the observed mean log(RT) for cooperation (blue) and defection (red) decisions as a function of cooperation beliefs, and their size represents number of observations. The predicted values from the regression Model 1 are represented by the dotted lines. As can be seen, RTs for cooperation and defection decisions follow opposite trajectories with respect to cooperation beliefs, so that the higher the cooperation beliefs, the faster the RTs for cooperation decisions and the slower the RTs for defection decisions.

After confirming the anticipated effect of expected payoffs on RTs, we now turn to the role of cooperation beliefs. We expect mean RTs for both cooperation and defection decisions to be strongly dependent on cooperation beliefs but, critically, that the speed of each response type will follow opposite trends as a function of cooperation beliefs. Figure 3 plots the observed mean log(RT) as a function of beliefs regarding the co-player’s cooperation intentions, together with the linear prediction of Model 1. The RT-slopes of cooperation and defection decisions across the levels of cooperation beliefs are clearly in opposite directions. For cooperation decisions, the effect of cooperation beliefs on log(RT) is negative (*b* = −0.442, *p* < 0.001), while for defection decisions it is positive (*b* = 0.217, *p* < 0.001). Defection decisions are faster under low cooperation beliefs but, as beliefs about partner’s cooperation intentions increase, defection decisions gradually become slower. The opposite holds for cooperation decisions. Cooperation is slow at low cooperation beliefs, but response latency speeds up as cooperation beliefs increase. In fact, despite the current experimental context was overall favorable for defection choices, in line with our prediction, when cooperation beliefs take the highest value (100%) response latencies for cooperation decisions are significantly faster than those for the defection decisions.

Note, that the effect of cooperation beliefs is not only statistically significant but also large in magnitude for both cooperation and defection choices even after controlling for similarity of options in monetary terms (a variable that already includes the probability of cooperation: dEV = payoffs × cooperation beliefs). Thus, the effect observed here is the *net* effect of the immediate social environment, as shaped by beliefs regarding the other’s cooperation intentions. Additionally, while the effect of dEV is similar across choices (slower RT for higher values of dEV in absolute terms), the magnitude of the effect of cooperation beliefs is not only different for cooperation and defection choices, but it goes in opposite directions. In other words, beliefs about others’ cooperation intentions would seem to function like a switch that determines when cooperation or defection is the fast choice. Therefore, while both the expected payoffs and the cooperation beliefs affect RTs, cooperation beliefs are the fundamental factor defining RTs in this social dilemma.

It is also worth noting that despite the general pattern described above, the relationship between cooperation beliefs and RTs (for both cooperation and defection decisions) displays a non-linearity at cooperation beliefs = 50%, which represents the highest uncertainty regarding the partner’s intentions. Under this uncertainty condition, defection decisions speed up above what would be expected from the linear trend, and even compared with conditions of less likely cooperation (cooperation beliefs = 20%). This non-linearity was not anticipated in our initial hypothesis, but one might speculate that it reflects aversion to uncertainty, making defection the default choice in this particular case (see^47,48^). Note however that, as discussed, the effect of cooperation beliefs on RTs does not simply reflect uncertainty, given the clear opposing tendencies in the zero uncertainty cases (cooperation beliefs = 100% and cooperation beliefs = 0%) as a function of choice made (for cooperation beliefs = 0%, defection decisions are the fastest whilst cooperation decisions are the slowest).

Once the role played by beliefs regarding others’ intentions has been settled, we turn the focus on how individual differences might affect RTs further. As discussed above, it is well-established that individuals differ in their propensity toward cooperation: while some are ready to cooperate in a given situation as long as they assume the same from their partners (i.e. they are conditional cooperators), others tend to defect, or “free-ride” on others’ cooperative behaviour (see^49^ for a review of the literature). We hypothesized that beliefs regarding a co-player’s cooperative intentions might affect the behaviour of these two individual profiles differently. Whereas the expectation that others are likely to cooperate will enhance cooperation propensity in conditional cooperators, the same expectation will have either null effects or even increase free riders’ propensity to defect, as they consider this as an opportunity for maximizing personal gains^50^. Additionally, in much the same way that such individual preferences moderate the effect that beliefs have on *behaviour*, in the current dataset individuals’ preferences might moderate the *speed* of each kind of behaviour under varying values of cooperation beliefs. In particular, for conditional cooperators high cooperation beliefs may make cooperation faster, just like it has been shown for the overall group. Instead, this might not work for free riders, for whom high cooperation beliefs may induce speed costs for the otherwise fast defection.

We estimated individual cooperation profiles from participants’ behavioural patterns in the game. In particular, we measured individuals’ choice probability to cooperate as a function of the co-player’s cooperation likelihood, i.e. as a proxy for the individual’s degree of conditional cooperativeness. We quantify this tendency by running a separate logistic regression on the proportion of cooperation choices for each participant across all rounds, using the cooperation belief (0, 20, 50, 80, and 100%) as the sole independent variable (100 observations per participant). Note that this regression is based on choice behaviour, not on RTs. The resulting coefficient, our measure for individual-level responsiveness to cooperation beliefs about the co-player (variable IndCoop), was subsequently entered as an individual-level variable into our basic model estimating log(RT) (see^51,52^ for similar methodologies).

As a first step, we introduced IndCoop and its interaction with choice type (cooperation or defection, a binary variable named Coop) in the model. This allows us to test whether individuals with different cooperation profiles differ in their speed of cooperation and defection choices (Model 2). Then, in order to test whether the *effect* of cooperation beliefs on RTs is different among individuals with different cooperation profiles we introduced the interactions Beliefs*IndCoop and Beliefs*IndCoop*Coop in a separate model (Model 3). At the same time, to test whether a moderation effect also exists with respect to the effect similarity of options in monetary terms has on RTs, we added in the same model the corresponding interactions, i.e. dEV*IndCoop and dEV*IndCoop*Coop.

From Model 2 we see that the more conditionally cooperative participants are, the faster they make their cooperation decisions (*b* = −0.664, *p* < 0.001) and slightly slower their defection decisions (*b* = 0.105, *p* = 0.062) (Figure S1). Importantly, from Model 3, we see that Beliefs*IndCoop (*b* = 0.370, *p* = 0.001) and the triple interaction Beliefs*IndCoop*Coop (*b* = −1.521, *p* < 0.001) are both highly significant, meaning that the effect of cooperation beliefs on response times is moderated by individual differences in cooperation attitudes. On the other hand, we observe only a marginal trend for the interaction effect between dEV and IndCoop (dEV*IndCoop: *b* = 0.029, *p* = 0.065; dEV*IndCoop*Coop: *b* = −0.060, *p* = 0.205), meaning that individual differences in cooperation attitudes do not have the same moderation effect on dEV-effect on RTs. In order to visualize the differential effect of cooperation beliefs on RTs, we classified participants into conditional cooperators and free riders and then replotted the interaction between cooperation choice and cooperation beliefs (previously shown in Fig. 3) now separately for each group. Conditional cooperators (24 out of 46 individuals, or 52% of our sample) were participants whose coefficient from the individual-level regressions of Beliefs over Coop was positive and highly significant (*p* < 0.002 for all but three individuals, for whom *p* was 0.018, 0.02 and 0.021), whereas free riders (the remaining 48% of the sample) were participants with either negative or positive but not statistically significant coefficient at conventional levels (*p* > 0.1 for all but three individuals, for whom *p* was 0.065, 0.071 and 0.073; see Figure S3a,b).

**Figure 4:**
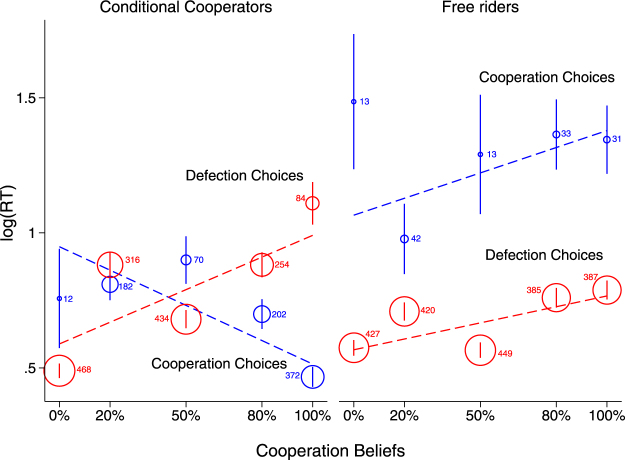
Social preferences as a moderator of the effect of cooperation beliefs on RTs. The figure presents the effect of cooperation beliefs on cooperation (blue lines) and defection (red lines) separately for conditional co-operators (left panel) and free riders (right panel). The predicted values (dotted lines) come from the regression Model 3. For conditional cooperators, cooperation and defection decisions follow opposite trajectories with respect to cooperation beliefs, so that the higher the cooperation beliefs, the faster the RTs for cooperation decisions and the slower the RTs for defection decisions. Among free riders, higher cooperation beliefs lead to higher RTs for defection decisions – just like conditional cooperators – as well as for their cooperation decisions (not statistically significant though).

Therefore, in Fig. 4, we plot the mean log(RT) separately for conditional cooperators and free riders against cooperation beliefs, as well as the linear prediction of Model 3. Figure 4 illustrates the differential effect of beliefs for the two typologies of individuals. For conditional cooperators, the relative speed of cooperation and defection choices depend heavily on beliefs; higher cooperation beliefs regarding the co-player make defection choices slower (*b* = 0.375, *p* < 0.001), while they speed up cooperation choices (*b* = −0.452, *p* < 0.001). Among free riders on the other hand, cooperation choices are slower than defection choices (*p* < 0.001) independently of beliefs, and higher cooperation beliefs increase RTs for both defection (*b* = 0.177, *p* < 0.001) and cooperation choices (*b* = 0.166, *p* = 0.305) even if in the latter case it does not reach significance due probably to the small number of observations. The interesting thing here is that higher cooperation beliefs make defection choices significantly slower even among free riders (like conditional cooperators), suggesting that a cooperative social environment induces speed costs universally among all individuals. Therefore, the general pattern observed in Fig. 3 holds for all individuals with respect to the negative effect of high cooperation beliefs on defection choices, while the positive effect of high cooperation beliefs on cooperation choices reflect the pattern observed among conditional cooperation, where the bulk of observations comes from.

Focusing on the effect of expected payoffs on mean log(RT), the picture is different. The two groups display the same RT pattern for both cooperation and defection decisions and what is more, the effect of dEV is now considerably smaller. Regarding cooperation decisions, the effect of dEV is positive for both conditional cooperators (*b* = 0.023, *p* = 0.012) and free riders (*b* = 0.040, *p* = 0.093), and again only marginally significant for the latter due probably to the small number of observations. Similarly, for defection decisions, the effect is negative and small among both conditional cooperators (*b* = −0.008, *p* = 0.269) and free riders (*b* = −0.023, *p* < 0.001). Therefore, we see that while similarity in terms of monetary returns drive RTs up among all individuals, its effect is small.

As a robustness test with respect to the measure of individual cooperation propensities used, we have replicated the analysis using a different proxy for cooperation tendencies. This is because albeit in principle an individual could be unresponsive to cooperation beliefs regarding the other players’ intentions, she could still be very cooperative, for example as an unconditional altruist (note, however that was not the case for none of the 22 individuals classified as free riders; see Figure S3b in the SI). Therefore, in defining the alternative proxy, we employed the probability of cooperation across the rounds for each individual indexed as the number of times out of 100 the individual choses to cooperate. Results remained essentially the same (see Tables S2 and S3 and Figures S2 and S3).

## Discussion

Overall, the results of the present study suggest that the social context created by beliefs regarding the partner’s intention to cooperate has an important impact on determining the speed of cooperation and defection decisions. High cooperation beliefs create a cooperative immediate social environment for conditional cooperators where cooperation decisions are fast at the expense of steep speed costs for defection decisions. In other words, the pattern of RTs as a function of cooperation beliefs display opposing slopes for cooperation and defection decisions. What is more, high cooperation beliefs slow down defection choices even among free riders whose social preferences profile would suggest defecting (i.e. taking advantage of others’ cooperative intentions) to be their unequivocal, fast choice.

These results stress the role of beliefs over monetary payoff, the role of which was drastically reduced when beliefs and social preferences were jointly accounted for in the analysis. More importantly the results indicate that cooperation beliefs do not only affect RTs in a direct way (like monetary payoffs), but they actually have a profound modulatory role. This *defining* effect of beliefs on cooperation and defection RTs for both conditional co-operators and free riders has important implications for the conflict of choice account, which in turn calls for reconsidering the intuitive cooperation hypothesis.

According to the conflict of choice account, RTs merely reflect how difficult is the choice in hand, which depends on how similar the available options are; the more similar the available options, the more difficult to reach a decision and ultimately, the higher the RTs. As such, conflict of choice includes monetary payoffs, social preference and beliefs. And while it is true that all these factors affect the relative attractiveness of the options, we suggest that employing such an all-inclusive definition, where beliefs are yet another additive component of the term, obscures their more fundamental role. Cooperation beliefs set the immediate social context and prime or define which behaviour is appropriate in each case: Cooperation in a cooperative social context and defection in a defection social context. Deviation from this pattern comes at a “cost” (resulting in longer RTs). Importantly, according to this proposed framework, RTs do not only reflect choice difficulty, but they might reflect that some behaviours in specific social contexts are *default* and not just *easy*, as suggested by the conflict of choices account.

Within the proposed framework, the critical role that beliefs play in choice RTs may have implications for the intuitive cooperation hypothesis. According to this hypothesis, cooperation has evolved as an intuitive impulse among humans and it is overridden only when they employ a deliberate reasoning mode that typically favours selfish outcomes^7,53^. Even if one cannot unequivocally induce intuition from fast choices in correlational studies such as the present one^29–32^, observing that the speed of cooperation is so much dependent on social beliefs about others’ cooperativeness points to *reciprocity*, rather than *cooperativeness* per se, as the potential ‘intuition’ at play^34^. In other words, in social dilemma situations, intuition does not unequivocally lead to either cooperation or defection. It seems that the intuitive thing to do is look into others’ cooperation intentions.

Remarkably, considering the effect of beliefs regarding others’ inclination to cooperate on RTs may provide a key in order to reconcile previous, ostensibly contradictory results in the literature. Consider for example the contradictory results of^5^ and^11^ which reported, respectively, that cooperation RTs were faster or slower (respectively) than defection RTs in the context of a very similar Public Good game. Apart from other design differences, importantly in^5^ (but not in^11^) participants engaged in a group activity before playing the game (either team work, cheap talk communication, mutual introduction or a combination). It is well-documented that these kinds of social interactions not only affect cooperative behaviour, but also beliefs about cooperative behaviour of others (see the seminal work by^54^). It is therefore plausible to assume that the cooperation beliefs were higher in^5^ than in^11^. Then, according to our framework, this difference has affected the social environment and therefore the relative speed of cooperation vs. defection choices. Thus, we might postulate that in both cases, the faster decisions were the default given the social context, namely cooperation in the case of^5^ and defection in the case of^11^.

Similarly, in both^6^ and^9^ participants played a Dictator game both as proposers and as responders. The former study, testing participants from Denmark, reported that the most pro-social choices were faster than the more selfish ones, whereas the latter, testing participants from Spain, reported the opposite pattern. However, interpersonal trust is well known to vary from country to country, and it happens to be higher amongst Danes than amongst Spaniards^55^, and thus the former might reasonably hold higher cooperation beliefs about others than the latter. Therefore, given our proposed framework and that in both cases the design allowed trustworthiness to affect decisions (participants played both roles and that was common knowledge), it is plausible to assume that cooperation beliefs (high for the Danes, low for the Spaniards) dictated the fastest choices in all cases (cooperative for the Danes and selfish for the Spaniards).

The same framework can be used to interpret studies suggesting intuition. For example^56^, found that cognitively depleted participants returned higher amounts in response to trusting (vs. untrusting) individuals in a Trust game, which point to reciprocal intuitiveness. In other words, cooperation was the intuitive behaviour whenever the individuals were holding high cooperation beliefs for the matched trustors, but not otherwise.

## Methods

### Participants and procedures

Students from Pompeu Fabra University (UPF) were recruited via e-mail (N = 50, average age 22.32 years old; 48% female), from a participants’ database. All of them were native Spanish speakers, or Spanish-Catalan bilinguals. The experiment was conducted in the CBC Lab of UPF, lasted approximately 1 hour, and 6 or 8 participants were tested in each session. Despite they were tested in individual booths to prevent visual contact or any communication between them, they could see each other in the waiting room, prior to the test. They were paid in cash at the end of the session in private (mean payoff was €10,0 including a show-up fee of €3). All methods were carried out in accordance with the Declaration of Helsinki, under a protocol approved by the UPF Ethics committee of the University of Pompeu Fabra (CEIC - Parc de Salut Mar). All Participants provided written informed consent prior to their participation. The protocol was custom programmed using Psychotoolbox libraries (running in Matlab R2015B).

### Prisoner’s Dilemma game

Participants played a discrete version of the Traveller’s Dilemma game^57^, which is in essence a Prisoner’s Dilemma game. The game was presented as a coordination game, whereby two players are asked to choose between two prices, low and high. If both participants choose the same price, either low or high, then they both end up with a payoff equal to that price. However, if one player chooses low and the other high, then the one who chooses low ends up with a payoff equal to the low plus a “reward”, which is represented by a third price (such that the sum of the reward and the low price exceeds the high price). The one who chooses the high price, ends up with a payoff that equals the low price minus a “penalty”, which is equal to the “reward”, i.e. the third price. Therefore, the high price corresponded to cooperation and the low price corresponded to defection. The social welfare was maximized if both participants chose the high price (cooperated) but choosing the low price (defection) always exhibited individual higher expected monetary payoffs, irrespectively of the partner’s decision (an English translation of the instructions is available in the Supplementary Information).

### Cooperation beliefs, decisions and RT recording

Subjects played a total of 100 rounds, in addition to 5 practice rounds. They were led to believe that they were playing against each other, even though there were no real opponents in the game. Please note that, to confer credibility to the story and lead them to believe they were playing against each other, participants were brought in the laboratory in groups of 6 to 8, before proceeding each to one individual testing booth. Participants played against pre-defined opponents’ strategies. At the beginning of each round participants received a visual cue about the likely strategy adopted by their partner assigned in that round represented with a graphic display of the ratio of trials the partner participant had chosen to cooperate over 20 rounds in the game, similar to a battery charge indicator (see Fig. 1). Following the cue, a payment structure was shown for her decision. Both the cue for cooperation beliefs and the payment structure were selected randomly without replacement from the pre-defined payment matrix, on a trial by trial basis. Cooperation beliefs could be 0% (partner never cooperates), 20%, 50%, 80% or 100% (partner always cooperates) with equal probability. That is, each participant faced a total of 20 rounds for each cooperation belief level shown randomly across the 100 rounds. RTs were recorded from the moment the payment structure was displayed on the screen until a response key associated to a decision was pressed. One round was randomly selected at the end of the game to determine payoffs.

### Individual measures of cooperativeness

We classify participants on the basis of their pattern of cooperation behaviour across rounds with varying cooperation beliefs, i.e. with respect to the probability of the other participant cooperating. To quantify participants’ tendency of increasing their probability of cooperating as a response to higher cooperation beliefs, we entered the variable Beliefs in a logistic regression as the independent variable, with the dependent variable being participants’ choice (cooperation or defection). The estimated coefficient for each participant was the measure of the individual’s responsiveness to cooperation beliefs. Based on these coefficients, participants were classified into two categories: conditional cooperators, those whose probability of cooperation was an increasing function of cooperation beliefs (see Figure S3a); and free riders, those who irrespectively of the cooperation beliefs of the other participant practically never cooperated (Figure S3b). The coefficient of these participants was always insignificant (*p* > 0.065). Alternatively, we measured cooperative tendencies measuring the probability of the individual cooperating in a given round. This probability then entered as an individual level variable [0, 0.68] into our basic model estimating log(RT). Regression analysis gave essentially identical results (see Tables S1 and S2 and Figures S1 and S2).

A note is in order here. It is well known that individuals with different social preferences profile have different beliefs regarding others’ cooperative behaviour. In particular, pro-social individuals tend to have higher cooperation beliefs than pro-self individuals (see^58^ and^59^ for a recent meta-analysis). Therefore, manipulating, rather than measuring beliefs is necessary to accurately measure the effect of beliefs on RTs, avoiding endogeneity. On the other hand, however, such an analysis excludes the potential effect that social preferences may have on beliefs. Additionally, by eliciting social preferences from choice behaviour (as in^51^ and^52^), rather than directly measuring them (employing for example the Social Value Orientation test for classifying individuals’ social preferences) there is a potential overlap between defection / cooperation choices and the classification of individuals as conditional cooperators and free riders.

### Statistical analysis

We conducted a panel data analysis that takes each participant as the unit and each round as time. A Breusch-Pagan test confirmed the superiority of a random effects model over a pooled OLS one (*p* < 0.001), and the Hausman test confirmed the superiority of a random effects model over a fixed effects model (*p* = 0.1262), giving equally consistent but more efficient estimators. Hence, we used a random effects model with both within-subject and between-subject variables, making sure that each observation is clustered at the individual level. After excluding incomplete observations, and one outlier (RT > mean RT + 3sd), we were left with 4600 observations (46 participants × 100 decisions). Our model estimates log(RT). The natural logarithm is used due to the heavily right-skewed decision time’s data^60^. The basic predictors are cooperation beliefs (variable Beliefs) and expected payoffs, the latter indexed as the difference in expected value terms (Beliefs × payoffs) between the chosen and the unchosen option, which we denote as dEV. Coop is included in the model as a binary variable taking the value of 1 if the choice has been cooperation and 0 if the choice has been defection. We expect by hypothesis that cooperation and defection choices will follow opposite trends with respect to cooperation beliefs, hence we also include the corresponding interaction term (Coop x Beliefs). The interaction of Coop with dEV is also included because dEV takes positive values for defection decisions and negative values for cooperation decisions, since by design, defection had always higher expected value than cooperation. Therefore, for defection choices it will translate in a negative coefficient of dEV on log(RT) and in a positive one for cooperation choices. Lastly, two further terms are used to capture well known sources of variability: sequential variations in RT across the experiment (variable Round, indicating the trial position in the experiment sequence, from 1 to 100), and individual differences in response latency (variable IndRT_i_, measured as the mean average RT of each individual across all 100 decisions; see^61^). In sum, we estimated the following model (1):

1 $$\begin{array}{rcl}log{(RT)}_{it} & = & {{\rm{\beta }}}_{0}+{{\rm{\beta }}}_{1}Belief{s}_{t}+{{\rm{\beta }}}_{2}dE{V}_{t}+{{\rm{\beta }}}_{3}Coo{p}_{t}+{{\rm{\beta }}}_{4}Belief{s}_{t}\times Coo{p}_{t}\\  &  & +\,{{\rm{\beta }}}_{5}dE{V}_{t}\times Coo{p}_{t}+{{\rm{\beta }}}_{6}Roun{d}_{t}+{{\rm{\beta }}}_{7}IndR{T}_{i}+{u}_{i}+{\varepsilon }_{it},\end{array}$$

where *i* indexes participants, *t* indexes decision choices and *u*_*i*_ and *ε*_*it*_ are the between- and within-participants error terms respectively. To analyze the potential effect of individual characteristics we added in the model the individual level variable IndCoop_i_ denoting the individual tendency to response to difference in cooperation beliefs. Therefore, we estimated a second model where we included IndCoop_i_ and its interaction with Coop_t_ as predictors of log(RT)_it_. Therefore, we estimated the following model (2):

2 $$\begin{array}{rcl}log{(RT)}_{it} & = & {{\rm{\beta }}}_{0}+{{\rm{\beta }}}_{1}Belief{s}_{t}+{{\rm{\beta }}}_{2}dE{V}_{t}+{{\rm{\beta }}}_{3}Coo{p}_{t}\\  &  & +\,{{\rm{\beta }}}_{4}Belief{s}_{t}\times Coo{p}_{t}+{{\rm{\beta }}}_{5}dE{V}_{t}\times Coo{p}_{t}+{{\rm{\beta }}}_{6}IndCoo{p}_{i}\\  &  & +\,{{\rm{\beta }}}_{7}IndCoo{p}_{i}\times Coo{p}_{t}+{{\rm{\beta }}}_{8}Roun{d}_{t}+{{\rm{\beta }}}_{9}IndR{T}_{i}+{u}_{i}+{\varepsilon }_{it}\end{array}$$

In a third model, we explored the role of IndCoop_i_ as a potential moderator of the effect of cooperation beliefs on log(RT)_it_ and thus we included the interaction terms Beliefs_t_ × IndCoop_i_ and Beliefs_t_ × IndCoop_i_ × Coop_t_. Additionally, we also included the same interaction terms regrading dEV: dEV_t_ × IndCoop_i_ and dEV_t_ × IndCoop_i_ × Coop_t_. In particular, we estimated the following model (3):

3 $$\begin{array}{rcl}log{(RT)}_{it} & = & {{\rm{\beta }}}_{0}+{{\rm{\beta }}}_{1}Belief{s}_{t}+{{\rm{\beta }}}_{2}dE{V}_{t}+{{\rm{\beta }}}_{3}Coo{p}_{t}\\  &  & +\,{{\rm{\beta }}}_{4}Belief{s}_{t}\times Coo{p}_{t}+{{\rm{\beta }}}_{5}dE{V}_{t}\times Coo{p}_{t}+{\beta }_{6}IndCoo{p}_{i}\\  &  & +\,{{\rm{\beta }}}_{7}IndCoo{p}_{i}\times Coo{p}_{t}+{{\rm{\beta }}}_{8}Belief{s}_{t}\times IndCoo{p}_{i}\\  &  & +\,{{\rm{\beta }}}_{9}Belief{s}_{t}\times \,IndCoo{p}_{i}\times Coo{p}_{t}+{{\rm{\beta }}}_{10}dE{V}_{t}\times IndCoopi+{{\rm{\beta }}}_{11}\\  &  & \times \,dE{V}_{t}\times IndCoo{p}_{i}\times Coo{p}_{t}+{{\rm{\beta }}}_{12}Roun{d}_{t}+{{\rm{\beta }}}_{13}\,IndR{T}_{i}+{u}_{i}+{\varepsilon }_{it},\end{array}$$

Analyses were performed with STATA 14 statistical software.

### Data availability

The dataset generated and analysed during the current study are available from the corresponding author on reasonable request.

## Electronic supplementary material

Supplementary Information

Do Code
