# Supplementary material for: Beliefs about others’ intentions determine whether cooperation is the faster choice
Source: Sci Rep. 2018 May 14;8:7509. doi: 10.1038/s41598-018-25926-3 (PMC5951794; doi:10.1038/s41598-018-25926-3)
Supplement: Supplementary file 1 — Supplementary Information [file 41598_2018_25926_MOESM1_ESM.pdf]

Supplementary Information for:

Beliefs about others' intentions determine whether cooperation is the faster choice

Juana Castro Santa, Filippos Exadaktylos & Salvador Soto-Faraco

Contents

Table S1 *Determinants of log(RT)*.. 2

Table S2 *Determinants of log(RT) using alternative individual-level cooperation variable*................ 3

Figure S1 *Individual cooperation attitudes over RTs (coopertion – defection)*.................................. 4

Figure S2 *Individual cooperation attitudes (alternative variable) over RTs (cooperation - defection)*.4

Figure S3 *Percentage of cooperation over cooperation beliefs for (a) conditional cooperators and (b) free riders*.. 5

Experimental Instructions.. 6

Table S1. Determinants of *log(RT)*

Log(RT) predictors	Model 1	Model 2	Model 3
Beliefs	.217*** (.029)	.233*** (.029)	.152*** (.038)
dEV	-.018*** (.004)	-.018*** (.004)	-.025*** (.006)
Coop	.471*** (.073)	.770*** (.082)	.419* (.110)
Beliefs **x** *Coop*	-.660*** (.066)	-.590*** (.066)	.066 (.181)
dEV **x** *Coop*	.054*** (.010)	.050*** (.010)	.066* (.026)
IndCoop		.105+ (.056)	-.176 (.114)
IndCoop **x** *Coop*		-.770*** (.098)	.059 (.360)
IndCoop **x** *Beliefs*			.370** (.177)
IndCoop **x** *Beliefs* **x** *Coop*			-1.521*** (.331)
IndCoop **x** *dEV*			.029+ (.016)
IndCoop **x** *dEV* **x** *Coop*			-.060 (.047)
IndRT	.322*** (.012)	.316*** (.012)	0.314*** (0.012)
Round	-.004*** (.000)	-.004*** (.000)	-0.004*** (0.000)
Constant	.014 (.047)	-.009 (.050)	.060 (.055)
N	4600/46	4600/46	4600/46
X^2	1103.21***	1158.86***	1246.45***

Notes: Estimates from a random effects model regressions (groups: individuals). The model estimates the mean log(RT). Beliefs take the values of 0, .2, .5, .8, and 1. dEV is the difference in expected terms between the chosen and the unchosen option. Coop is a binary variable, taking the value of 1 for cooperation decisions and 0 for defection decisions. IndCoop is an individual level variable denoting the propensity to increase cooperation responding to higher cooperation beliefs; it is measured participants' behavioural patterns in the game (see text). IndRT is an individual level variable and stands for the mean RT of an individual across all 100 rounds. Round denotes the trial position in the experiment sequence, from 1 to 100. Standard error in parentheses. +p<0.1 * p<0.05, ** p<0.01, *** p<0.001.

Table S2. Determinants of *log(RT)* using alternative individual-level cooperation variable

Log(RT) predictors	Model 1	Model 2
Beliefs	.235***<br>(.029)	.132**<br>(.040)
dEV	-.018***<br>(.004)	-.026***<br>(.006)
Coop	.785***<br>(.083)	.813***<br>(.175)
Beliefs **x** *Coop*	-.670***<br>(.066)	-.437**<br>(.154)
dEV **x** *Coop*	.053***<br>(.010)	.105***<br>(.023)
IndCoop2	.237**<br>(.088)	-.230<br>(.187)
IndCoop2 **x** *Coop*	-.963***<br>(.125)	-.762+<br>(.427)
IndCoop2 **x** *Beliefs*		.702***<br>(.188)
IndCoop2 **x** *Beliefs* **x** *Coop*		-1.101**<br>(.394)
IndCoop2 **x** *dEV*		.047+<br>(.026)
IndCoop2 **x** *dEV* **x** *Coop*		-.169**<br>(.057)
IndRT	.315***<br>(.012)	0.314***<br>(0.012)
Round	-.004***<br>(.000)	-0.004***<br>(0.000)
Constant	-.021<br>(.050)	.065<br>(.058)
N	4600/46	4600/46
X^2	1181.44***	1118.28***

Notes: Estimates from a random effects model regressions (groups: individuals). The model estimates the mean log(RT). Beliefs takes the values of 0, .2, .5, .8, and 1. dEV is the difference in expected terms between the chosen and the unchosen option. Coop is a binary variable, taking the value of 1 for cooperation decisions and 0 for defection decisions. IndCoop2 is an individual level variable denoting the probability of cooperation; it is measured using the number of times, out of 100, that an individual chose to cooperate. IndRT is an individual level variable and stands for the mean RT of an individual across all 100 rounds. Round denotes the trial position in the experiment sequence, from 1 to 100. Standard error in parentheses. +p<0.1 * p<0.05, ** p<0.01, *** p<0.001.

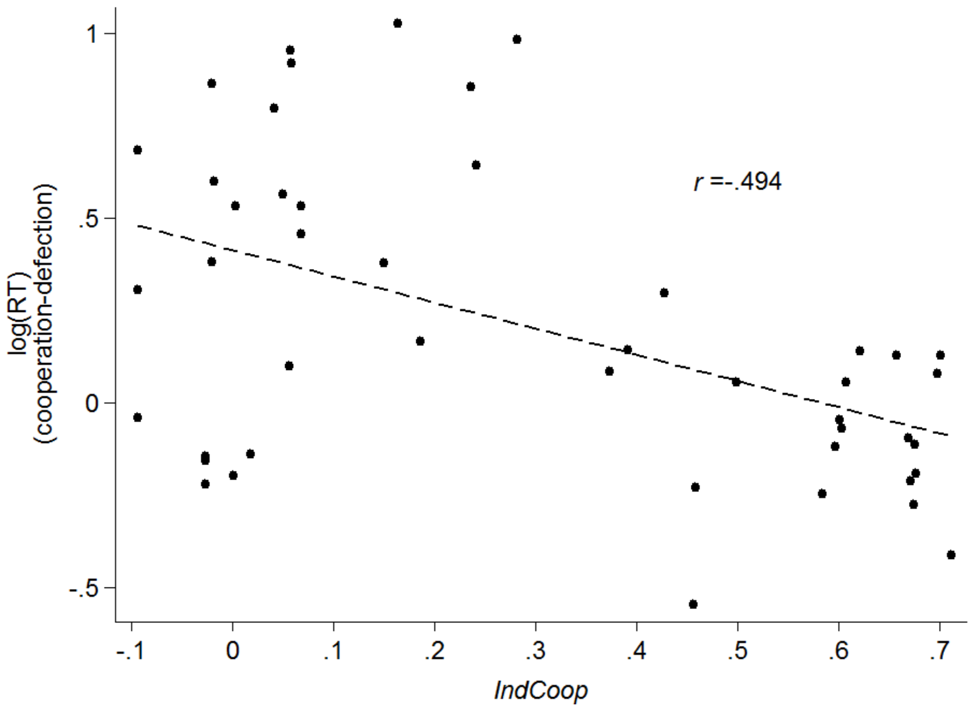

Figure S1. Individual-level responsiveness to cooperation beliefs (variable IndCoop) plotted against the difference between cooperation and defection RTs, at the individual level. Each dot represents an individual.

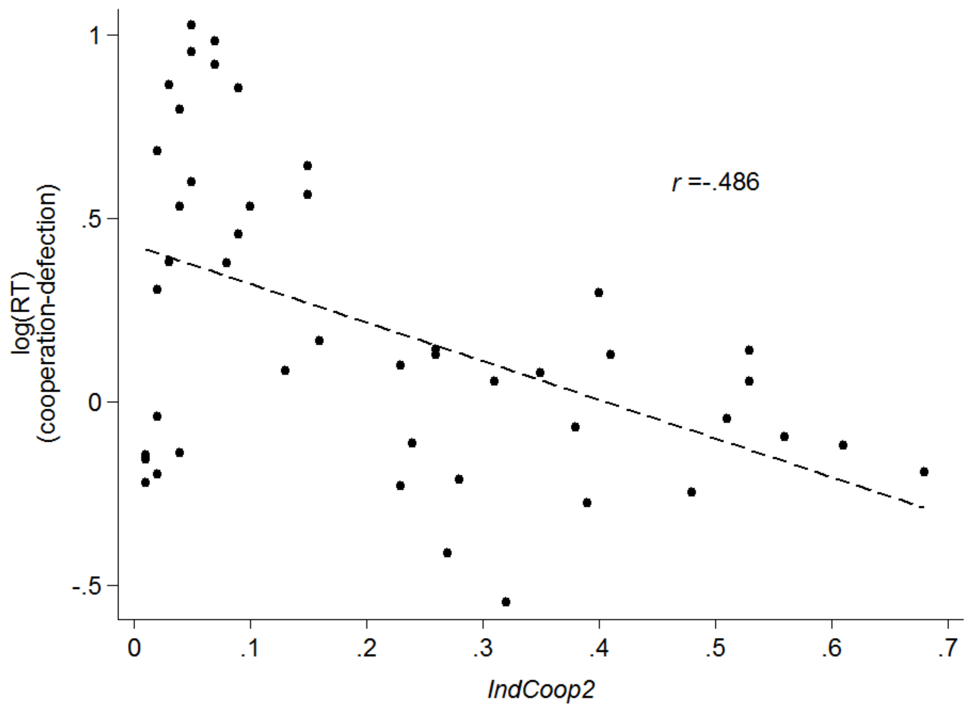

Figure S2. Probability of cooperation of each individual across the 100 rounds (variable IndCoop2) plotted against the difference between cooperation and defection RTs, also at the individual level. Each dot represents an individual.

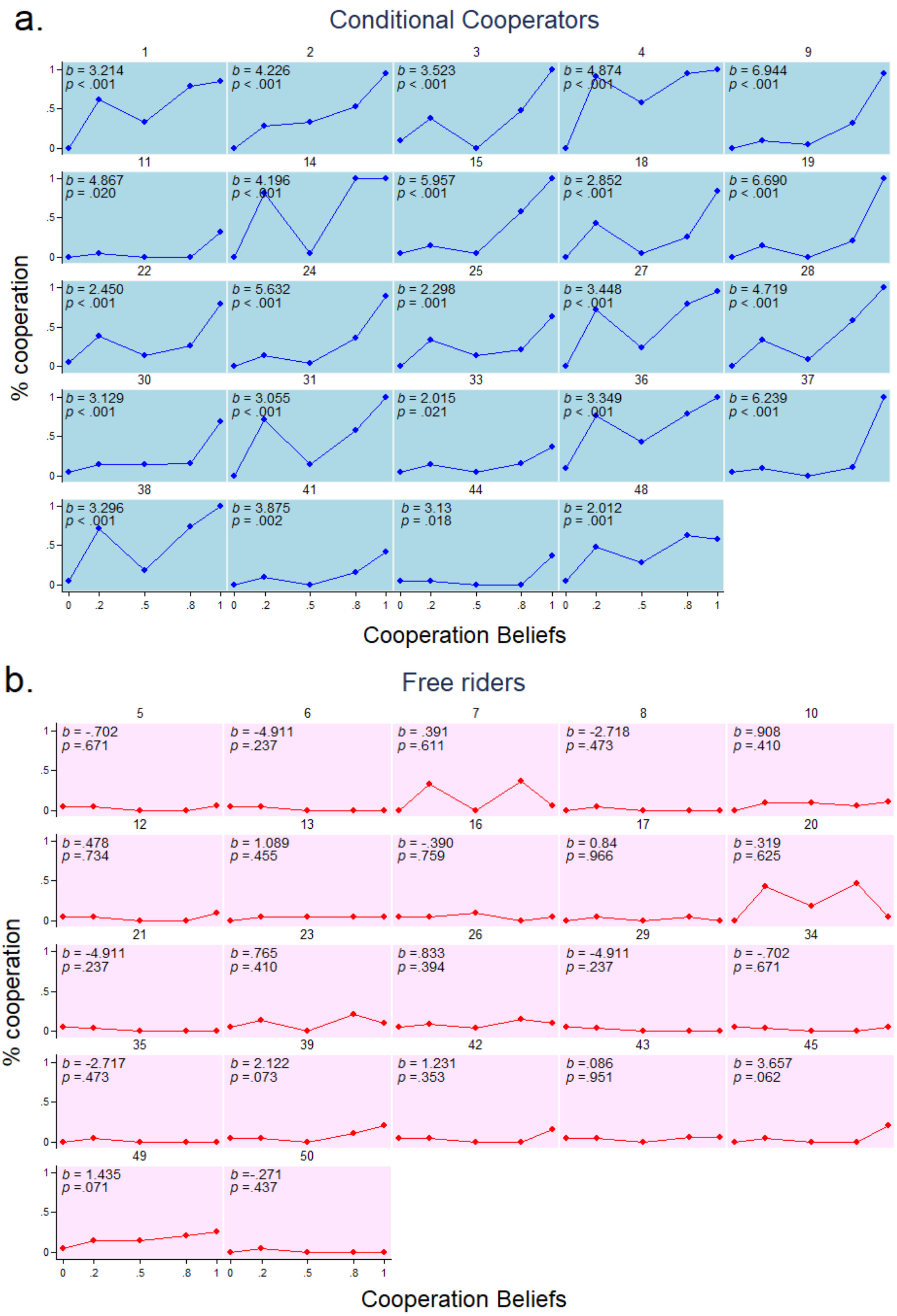

Figure S3. Percentage of cooperation over cooperation beliefs for (a) conditional cooperators and (b) free riders. For each participant, it is also depicted the coefficient and the *p* value of a logistic regression-one for each participant- across all rounds, with game decision (cooperate/defect) as the dependent variable and beliefs as the sole independent variable (100 observations per participant). Conditional cooperators are classified individuals with positive and statistically significant coefficient at 5%.

Experimental Instructions

You are participating in a Coordination Game.

As a show-up fee you have already earned 3 euros. Additionally, you could earn up to 20 euros more in this game depending on your decisions and those of other participants. Pay close attention to the instructions. The dilemma in this game is the following:

Two travelers come home from a remote island where they bought identical souvenirs. At their arrival, the passengers find out their souvenirs have been destroyed. The airline will have to pay a compensation equal to the price of the souvenir to the passengers. For the airline, it is impossible to know the exact price of the souvenir, nonetheless the airline has estimated an approximate range in which the price may fall. On the search for the honest price of the souvenir the airline separates the 2 travelers so they cannot confabulate, and asks them to report separately the price of the object.

If **both report the same price** it would be reasonable to assume that both are saying the truth and the **airline will reimburse this quantity** of money to each of the passengers.

If the passengers report different prices, the airline will take the **lowest price as the true cost of the souvenir** and each passenger will receive this amount as compensation.

Nevertheless, a discount on the amount of the compensation is going to be made to the passenger that reported the Highest Price as a <u>Penalty.</u> This amount will be given to the passenger that reported the Lowest Price as a <u>Reward</u> for his “honesty”.

In this coordination game, there are two types of Passengers: A and B. Each Passenger A will be randomly matched to play with a Passenger B.

Each round two different quantities of money that the airline is proposing to the passengers as compensation for the souvenir will be shown, as well as the amount of money the airline will impose as reward/penalty if two different prices of the souvenir are reported.

Each participant will take the decision separately on which quantity to report as the price of the souvenir. This procedure will be repeated for 100 rounds.

At the end of the game, one of the rounds will be randomly selected to be paid. The payment you will receive will depend on your decision and the one made by the other participant in the round chosen.

Example of Decision for one round

The airline offers the following amounts as compensation to the passengers:

(The amount of the reward/penalty appears in the center of the screen in red)

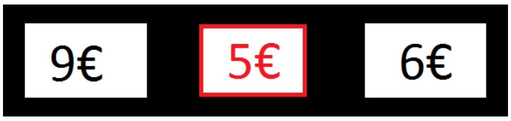

- If both passengers report that the souvenirs price was 9 euros, both receive 9 euros

- If both passengers report that the souvenirs price was 6 euros, both receive 6 euros

- If different prices are reported, both passengers will get the Lowest Price and additionally:

The passenger that reported the **Lowest Price receives a Reward**

Low Price (6 euros) + Reward (5 euros) = Receives 11 euros

The passenger that reported the **Highest Price pays a Penalty**

Low Price (6 euros) - Penalty (5 euros) = Receives 1 euro

Passengers A and Passengers B will take decisions in a different way.

Passengers A will define their strategy for a block of 20 rounds and Passengers B will define their strategy round by round.

You are reading the Instructions for Passenger B.

Passengers A will be asked to define a strategy for a block of 20 rounds by typing in the decision screen the number of rounds out of 20 in which they want to choose the High Price.

Passenger A will have to determine this decision for 20 rounds with no prior information. The computer will randomly assign one decision from the reported strategy to each round, and after 20 rounds they will be able to update their strategy.

Before going to the decision screen where you select the price you want to choose, you will be shown for 2 seconds the strategy reported by the Passenger A you were matched with to play the round. The strategy shows the number of times the Passenger A decided to pick the High Price of the souvenir in a green bar in the following way:

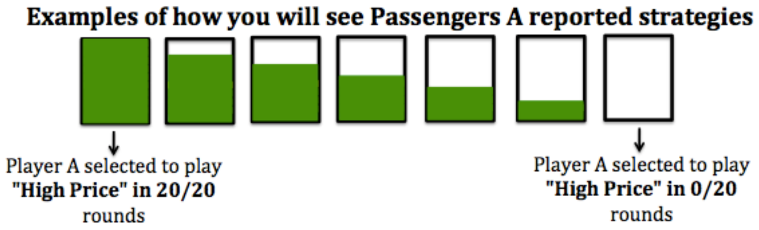

Each round you will be randomly matched with the Passenger A in the room and you will have 2 seconds to see his strategy.

Once you have seen Passenger A's strategy, you will be shown another screen with the two prices offered by the airline as well as the amount of the penalty. You will then select one of the two prices.

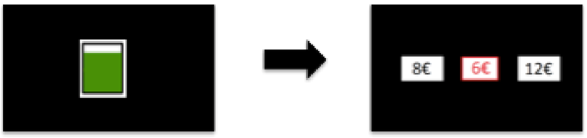

One of the 100 rounds will be randomly selected to be paid at the end of the game. Your earnings and those of the Passenger A assigned to play with you that round will depend on both your decisions.
